# Comparative Transcriptome Profiling of Ileal and Cecal Tissues Between Pekin Ducks and Shaoxing Ducks

**DOI:** 10.3390/genes16050488

**Published:** 2025-04-25

**Authors:** Dandan Wang, Zhengyu Hu, Ayong Zhao, Tao Zeng, Tiantian Gu, Wenwu Xu, Yong Tian, Lizhi Lu, Li Chen

**Affiliations:** 1 College of Animal Sciences and Veterinary Medicine, Zhejiang A&F University, Hangzhou 311300, China; wangddrich@163.com (D.W.); 17733388235@163.com (Z.H.); zay503@zafu.edu.cn (A.Z.); 2 Zhejiang-Ukraine Joint Laboratory for Poultry Germplasm Resources Conservation, Zhejiang Academy of Agricultural Sciences, Hangzhou 310021, China; zengtao4009@126.com (T.Z.); gtt19931029@126.com (T.G.); xuwenwu248@outlook.com (W.X.); tyong@zaas.ac.cn (Y.T.)

**Keywords:** cecum, duck, ileum, transcriptome

## Abstract

**Background**: Pekin ducks are well-known meat-type ducks, whereas Shaoxing ducks are bred for their egg-laying abilities. Growth and development of poultry species is well studied; however, very little is known regarding differences in intestinal gene expression between Pekin and Shaoxing ducks. **Methods**: To investigate intestinal differences between Pekin and Shaoxing ducks, we conducted transcriptome analysis on ileal and cecal tissues from five 42-day-old ducks per breed, raised under identical housing and feeding conditions to minimize environmental influences. **Results**: The results showed that a total of 379 differentially expressed genes (DEGs) with *p* < 0.05 and |log_2_FoldChange| > 1 were identified in the ileum when Pekin ducks were compared to Shaoxing ducks, among which 158 were upregulated and 221 were downregulated. Compared to Shaoxing ducks, a total of 367 DEGs with *p* < 0.05 and |log_2_FoldChange| > 1 were identified in the ceca of Pekin ducks, among which 204 were upregulated and 163 were downregulated. Among these DEGs, nine genes were reported to be associated with growth and metabolism, namely, *P2RX6*, *KCNJ6*, *CASQ2*, *EHHADH*, *ACSBG1*, *ELOVL4*, *AIF1L*, *VILL*, and *FABP1*. Functional enrichment analyses using the Gene Ontology (GO) and Kyoto Encyclopedia of Genes and Genomes (KEGG) databases indicated that the DEGs were significantly involved in pathways such as calcium signaling, unsaturated fatty acid biosynthesis, fatty acid degradation, and tryptophan metabolism. **Conclusions**: In conclusion, our study identified transcriptome differences in the intestines of meat-type and laying-type ducks, offering insights into the genetic basis of their growth and metabolic differences. Future studies should validate key genes and explore environmental influences on gene expression.

## 1. Introduction

Pekin ducks and Shaoxing ducks are two indigenous varieties in China, both of which are domesticated descendants from mallards [1]. Pekin duck is a world-famous breed of meat-type duck [2]. The famous Pekin duck, which originated in the western suburbs of Pekin near Yuquan Mountain, is now produced worldwide. It is known for its early maturity, fast growth rate, and strong fat deposition ability. Shaoxing duck is a high-yielding egg-laying breed in China [3]. It originated in Shaoxing, Xiaoshan, and other areas of Zhejiang Province. This breed is characterized by early maturation, high egg yield, low feed intake, and robust stress resistance—attributes that have led to its informal designation as “the pearl of poultry”.

The gut is crucial in poultry production, serving as a major part of the immune defense system and the main hub for feed nutrient breakdown and uptake [4]. Research indicates that pigeons’ wellbeing largely depends on efficient nutrient uptake in the small intestine. [5]. The avian ceca play a crucial role in hindgut nervous system development [6]. Poultry obtain the energy required for growth metabolism primarily from nutrient breakdown in the small intestine. The ileum is vital as the primary site for water and mineral absorption [7]. Its function is to promote the uptake of nutrients from digested food and deliver them into circulation, providing the organism with vital substances and energy. The cecum is a critical segment of the large intestine, located at the junction where the small and large intestines meet, and extending proximally along the small intestine. Its functions encompass microbial degradation of specific carbohydrates, reabsorption of water and salts, synthesis of certain vitamins by microbes, and breakdown of nitrogenous compounds [8]. There is a significant difference in growth metabolism between broiler ducks and laying ducks. Previous studies have shown that the weight growth curves of Pekin ducks aged 2 to 14 weeks are significantly greater than those of Shaoxing ducks [9]. Moreover, Pekin ducks exhibit a consistently faster growth rate than Shaoxing ducks during the entire period from 4 to 14 weeks of age [9]. Compared to the duodenum and ileum, the early jejunal development of waterfowl is most strongly correlated with body weight growth [10]. The miRNA expression profile of the small intestine in Shaoxing ducks under high-temperature conditions is primarily related to cell proliferation and metabolic processes [11]. Transcriptome analysis has uncovered genes linked to growth and metabolism in the ileum of Muscovy ducks, including seven genes, namely, *ANPEP*, *ENPEP*, *UPP1*, *SLC2A2*, *SLC6A19*, *NME4*, and *LOC106034733* [12]. These studies indicate that intestinal tissues significantly contribute to the growth and metabolic functions in ducks.

There are many reports on the intestinal microbiota of ducks, but few studies on the differences in intestinal tissue of ducks. While the primary intestinal functions and physiological differences in poultry are well-documented, understanding gene expression in intestinal tissues provides direct insight into the host’s genetic regulation of metabolism and nutrient absorption, which cannot be fully inferred from microbial analysis alone. Microbiota studies primarily reveal microbial composition and interactions, whereas transcriptome analysis allows for the identification of functional genes related to digestion, immune function, and metabolic pathways. Bridging this gap in knowledge is essential for comprehending how genetic factors influence intestinal function and overall duck physiology.

Based on previous research conducted by our group, significant differences in body weight and the morphology of the ileum and cecum were observed between 42-day-old Shaoxing ducks and Pekin ducks, yet the differences in their ileal and cecal transcriptomes remain unknown [13]. This study aims to explore the intestinal differences in the ileum and cecum between Pekin ducks and Shaoxing ducks under the same age and feeding conditions, as well as to pinpoint key differentially expressed genes (DEGs) associated with growth and metabolism in these intestinal tissues. By focusing on transcriptomic variations, we seek to provide a theoretical foundation for understanding the genetic regulation of growth and metabolism in ducks. The findings from this study have practical implications. Identifying genes related to intestinal function and metabolism can contribute to improving duck breeding strategies, optimizing feed formulation, and enhancing overall poultry production efficiency. This study expands the exploration of aspects of intestinal function in ducks, which may help to detect new and interesting functions from a biological perspective. Understanding these transcriptomic differences may help uncover novel regulatory mechanisms that contribute to breed-specific growth and metabolic traits, meriting further exploration in experimental and applied research.

## 2. Materials and Methods

### 2.1. Experimental Animals and Samples Preparation

In total, 122 male Shaoxing ducks and 160 male Pekin ducks were provided by the Guowei Poultry Industry Development Co., Ltd., Zhuji City, Shaoxing City, Zhejiang Province, China. The feeding trials were conducted from day 1 to day 42 of age. Each duck received a starter diet from day 1 to 3 weeks that was formulated with 18.7% crude protein and 12.81 MJ/kg of metabolizable energy (ME), followed by a grower diet from 4 to 6 weeks containing 17.1% crude protein and 11.67 MJ/kg ME. All ducks were raised under identical feeding and housing conditions to ensure consistency in the experimental environment. They were provided with a standard commercial diet formulated to meet their nutritional requirements at different growth stages. Feed and water were available ad libitum, and no additional dietary supplements were used.

At 42 days of age, 5 healthy ducks were randomly selected from each breeds. The ileum and cecum of Shaoxing ducks were labeled as SX_H and SX_M, respectively, while the ileum and cecum of Pekin ducks were both labeled as BJ_H and BJ_M. These ducks were fasted for 12 h before being weighed and slaughtered to reduce variability in gene expression caused by recent feeding. Simultaneously, the ileal and cecal tissues of the selected ducks were immediately collected after cleaning and removal of intestinal contents. The intestinal contents were removed, rinsed with PBS to inactivate RNases, washed multiple times, rapidly frozen in liquid nitrogen, and kept at −80 °C for transcriptomic analysis.

### 2.2. RNA Extraction, Library Construction, and Sequencing

RNA-seq libraries were constructed from 5 ileal and 5 cecal RNA samples. For each individual sample of ileal and cecal tissue, RNA was isolated via the TRIzol (Invitrogen Life Technologies) method, yielding total RNA. To prevent RNA degradation, RNase inhibitors were used during the extraction process, and all samples were kept on ice. The RNA concentration and purity were assessed using a NanoDrop 2000 spectrophotometer (Thermo Scientific, Waltham, MA, USA). The RNA was considered of good quality if the 260/280 ratio ranged from 1.8 to 2.0 and the 260/230 ratio exceeded 2.0. Samples exhibiting an RNA Integrity Number (RIN) of at least 7.0, as determined by the Agilent 2100 Bioanalyzer (Agilent Technologies, Santa Clara, CA, USA), were selected for the construction of sequencing libraries. mRNA containing poly(A) tails was subsequently isolated from the total RNA by employing Oligo (dT) magnetic beads. mRNA was sheared into approximately 300 bp fragments through ion-mediated fragmentation, as this fragment length was selected to balance sequencing efficiency and data quality. Shorter fragments increase the proportion of adapter sequences, reducing the effective data yield, whereas longer fragments may hinder cluster generation during sequencing. Random hexamer primers and reverse transcriptase were utilized to synthesize the first-strand cDNA, which subsequently served as a template for generating the second-strand cDNA. After cDNA synthesis, PCR amplification was performed to enrich the library fragments, and size selection was conducted to obtain a final library size of approximately 450 bp. The library fragment size was assessed using the Agilent 2100 Bioanalyzer. The total concentration of the libraries was determined using the PicoGreen assay (Quantifluor-ST Fluorometer, Promega, Madison, WI, USA, E6090; Quant-iT PicoGreen dsDNA Assay Kit, Invitrogen, Carlsbad, CA, USA, P7589), while their effective concentration was quantified by qPCR (StepOnePlus Real-Time PCR System, Thermo Fisher Scientific, Waltham, MA, USA).

Transcriptome libraries were subjected to high-throughput sequencing using the Hiseq X Ten system (Illumina, San Diego, CA, USA). Once raw data were obtained, Trimmomatic software (v0.39) was used to filter low-quality data of the original base sequences, and Cutadapt software (v3.4) was used to intercept sequencing adapter primers in order to obtain clean reads. A reference genome index was created using the Anas platyrhynchos genome (version ASM874695v1, file: anas_platyrhynchos.ASM874695v1.DNA.toplevel.fa), and HISAT2 (v2.1.0) was used to align paired-end clean reads to the reference genome.

### 2.3. Quality Control and Alignment of Raw Sequencing Data

Raw sequencing data were converted to FASTQ (http://en.wikipedia.org/wiki/FASTQ_format, accessed on 22 October 2024) format and subjected to quality control, including adapter trimming and low-quality read removal, followed by quality assessment using base quality and nucleotide composition distribution to ensure high-quality data for downstream analysis. The reference genome index was constructed using Bowtie2, and high-quality reads were aligned to the genome using TopHat2. The distribution of mapped reads across different functional regions of the genome, as well as their coverage uniformity along gene bodies, was subsequently analyzed to evaluate mapping quality and transcript expression characteristics.

### 2.4. Gene Expression Analysis

The raw indication of gene expression, the read count value, was quantitatively assigned to each gene using HTSeq (v0.9.1). FPKM (fragments per kilobase of transcript per million mapped reads) is a normalization method used in RNA sequencing to quantify gene expression levels. It accounts for both the length of the gene and the total number of reads in the sample, allowing for comparisons across different genes and datasets. Each fragment in paired-end sequencing is equivalent to two reads, and FPKM only counts fragments that map to the same transcript.

### 2.5. Differential Gene Expression Analysis

Differential gene expression analysis between the two comparison groups was conducted using the DESeq (v1.38.3) program, which employs a negative binomial distribution model to account for the overdispersion typically observed in RNA-seq count data. Subsequently, Pearson’s correlation analysis was performed to assess the relationships between gene expression levels, contingent upon the data distribution. Genes exhibiting significant differential expression were identified based on a *p* < 0.05 and an absolute |log_2_FoldChange| > 1. These criteria ensure that the detected changes are both statistically significant and biologically meaningful.

### 2.6. GO and KEGG Enrichment Analysis of Differentially Expressed Genes

We counted the number of DEGs in each term after mapping every gene to a phrase in the GO (Gene Ontology) (http://geneontology.org/, accessed on 22 October 2024) database. The top GO package (v2.50.0) was employed to carry out GO enrichment analysis on all DEGs, as well as on subsets of upregulated and downregulated DEGs. The hypergeometric distribution method was used to determine *p*-values(*p*), and a significant criterion of *p* < 0.05 was established. To identify the main biological functions linked to these genes, the GO terms with highly enriched differential genes were found. KEGG (UniProt Knowledgebase, http://www.uniprot.org/help/uniprotkb, accessed on 22 October 2024) pathway enrichment analysis for the differential genes was carried out using ClusterProfiler (v4.6.0), focusing on pathways with significant enrichment (*p* < 0.05). Additionally, all genes were also subjected to Gene Set Enrichment Analysis (GSEA) (v4.1.0), and pathway maps for GSEA enrichment were produced.

### 2.7. Real-Time Quantitative PCR (RT-qPCR) Analysis

The CFX384 real-time fluorescence system was utilized for RT-qPCR to verify the accuracy and reliability of RNA-seq gene expression data from ileum and cecum libraries of Pekin and Shaoxing ducks. The iScript cDNA Synthesis Kit (Bio-Rad Laboratories, Hercules, CA, USA) was used to create first-strand cDNA from 300 ng of RNA for each sample in a 4 μL reaction volume. The cDNA was diluted 10-fold, and the RT-qPCR reaction (10 μL) was set up as follows: 5 μL of Power SYBR^®^ Green Master Mix, 0.4 μL each of gene-specific forward and reverse primers (10 μmol/L), 2.2 μL of sterile water, and 2 μL of cDNA template. A 30 s initial denaturation at 95 °C was followed by 40 cycles of 95 °C for 5 s and 60 °C for 30 s (for gathering fluorescence data) as the reaction conditions. Using *GAPDH* as the internal reference gene, the 2^−ΔΔCt^ method was used to quantify the relative expression level of each gene in each sample, which was examined in triplicate [14]. Quantification primers were designed using the Primer-BLAST tool available on the NCBI website (https://www.ncbi.nlm.nih.gov/tools/primer-blast/, accessed on 11 November 2024). Detailed gene information for real-time PCR is provided in Table 1.

## 3. Results

### 3.1. Transcriptome Sequencing of the Ileum and Cecum from Pekin and Shaoxing Ducks

In this study, 10 libraries were established for high-throughput RNA sequencing using the ileum and cecum from Pekin ducks and Shaoxing ducks (Table 2.). The transcriptomes of ileum and cecum yielded approximately 446 million (44,615,115 on average) and 445 million (44,506,480 on average) clean reads, respectively, accounting for 94.54% and 94.53% of the original reads. In this study, the average Q30 value was 92.48%. The chosen experimental samples are dependable biological replicates, as evidenced by the Pearson correlation coefficient for gene expression levels between samples above 0.8 (Figure S1).

### 3.2. Differentially Expressed Genes (DEG) Between Pekin and Shaoxing Ducks

**Figure 1:**
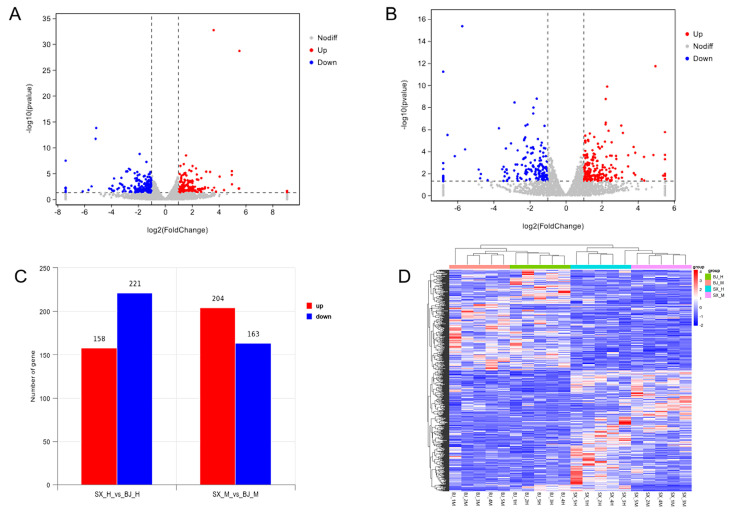
(**A**) Volcanic plots of total ileal gene expression in Pekin ducks and Shaoxing ducks. (**B**) Volcanic plots of total cecal gene expression in Pekin ducks and Shaoxing ducks. The x-axis indicates the log_2_ (fold change) value of gene expression, while the y-axis represents −log_10_(*p*-value) (where the *p*-value in the figure represents the *p*-value; *p* < 0.5 means −log_10_(*p*-value) > 1.0. Red dots indicate upregulated DEGs, green dots represent downregulated DEGs, and grey dots denote non-differentially expressed genes. (**C**) A bar chart showing the DEGs in the ileum and cecum of Pekin ducks and Shaoxing ducks, with red bars indicating upregulated genes and blue bars indicating downregulated genes. (**D**) Ileal and cecal tissue expression profiles of 616 DEGs in the SX vs. BJ group. The color scale in the heatmap represents the log_10_-transformed, FPKM-normalized expression values. Each horizontal bar corresponds to a gene, while each vertical column represents a sample, as indicated by the sample names. Red denotes upregulated genes, and blue signifies downregulated genes.

Using a threshold of *p* < 0.05 and |log_2_ FoldChange| > 1, differentially expressed genes (DEGs) were identified in both the ileum and cecum between Pekin ducks (experimental group) and Shaoxing ducks (control group). The volcano plots in Figure 1A,B depict the distribution of DEGs in the ileum and cecum, respectively. In the ileum, a total of 158 genes were significantly upregulated, and 221 genes were downregulated in Pekin ducks relative to Shaoxing ducks. Similarly, in the ceca, 204 upregulated and 163 downregulated genes were detected. The total numbers of DEGs in each tissue are summarized in the bar chart shown in Figure 1C. Furthermore, hierarchical clustering analysis based on the identified DEGs clearly separated individuals from the two duck breeds, demonstrating the robustness and consistency of the gene expression profiles (Figure 1D). A complete list of DEGs identified in the ileum and cecum between Pekin ducks and Shaoxing ducks is provided in Table S1 and Table S2, respectively.

### 3.3. GO Enrichment Analysis for DEGs

In order to further investigate DEG functions, GO enrichment analysis was conducted to annotate them and assess their functional distribution. There were 184 and 158 GO terms that were enriched for ileum and cecum DEGs, respectively (*p* < 0.05).

**Figure 2:**
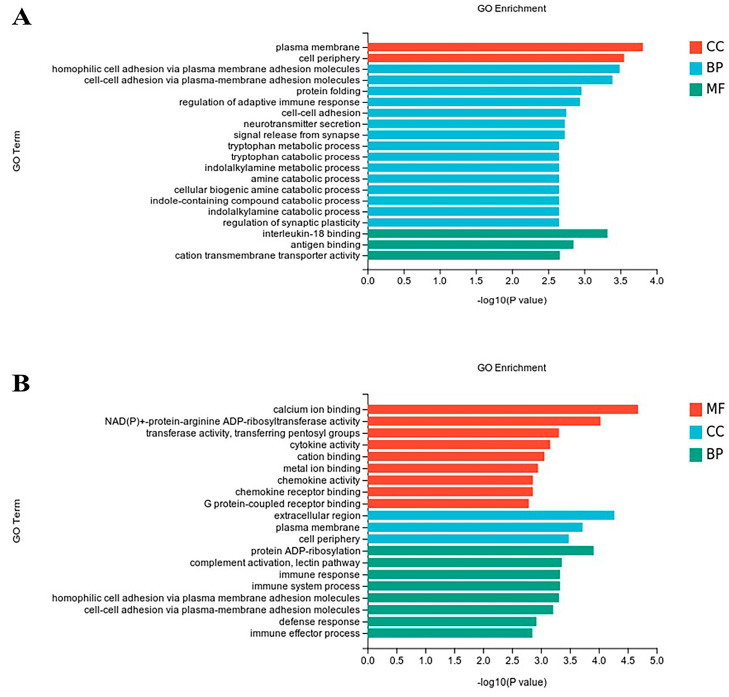
The bar chart of the top 20 GO terms enriched in DEGs. (**A**) The top 20 GO terms enriched in differentially expressed genes between Shaoxing ducks and Pekin ducks’ ileum. (**B**) The top 20 GO terms enriched in differentially expressed genes between Shaoxing ducks and Pekin ducks’ ceca.

The 20 most significantly enriched GO terms of ileum were displayed in Figure 2A. Cellular components contained 27 significance terms (*p* < 0.05), such as the plasma membrane, cell periphery, integral component of membrane, intrinsic component of membrane, and cell junction. And biological processes involved 113 significance terms (*p* < 0.05); among them, the 5 most enriched terms were homophilic cell adhesion via plasma membrane adhesion molecules, cilium or flagellum-dependent cell motility, cilium-dependent cell motility, cell–cell adhesion via plasma–membrane adhesion molecules, and protein folding. In total, 44 molecular function terms showed significant enrichment (*p* < 0.05), such as calcium ion binding, interleukin-18 binding, antigen binding, cation transmembrane transporter activity, somatostatin receptor activity, and ion transmembrane transporter activity. All GO enrichment terms identified from the ileal transcriptomes of Pekin and Shaoxing ducks are listed in Table S3.

The top 20 enriched GO terms of ceca were subjected to further analysis to identify their associated regulatory functions (Figure 2B). Molecular function contained 58 significance terms (*p* < 0.05), such as calcium ion binding, NAD(P)+-protein–arginine ADP-ribosyltransferase activity, transferase activity, transferring pentosyl groups, cytokine activity, and cation binding. And the cellular component involved 14 significance terms (*p* < 0.05); the top 5 terms included extracellular region, plasma membrane, cell periphery, dystroglycan complex, and sarcoglycan complex. In total, 86 terms (*p* < 0.05) were significantly enriched in biological process, for example, interspecies interaction between organisms, protein ADP-ribosylation, immune response, immune system process, and complement activation, lectin pathway. All GO enrichment terms identified from the cecal transcriptomes of Pekin and Shaoxing ducks are listed in Table S4.

### 3.4. KEGG Enrichment Analysis for DEGs

**Figure 3:**
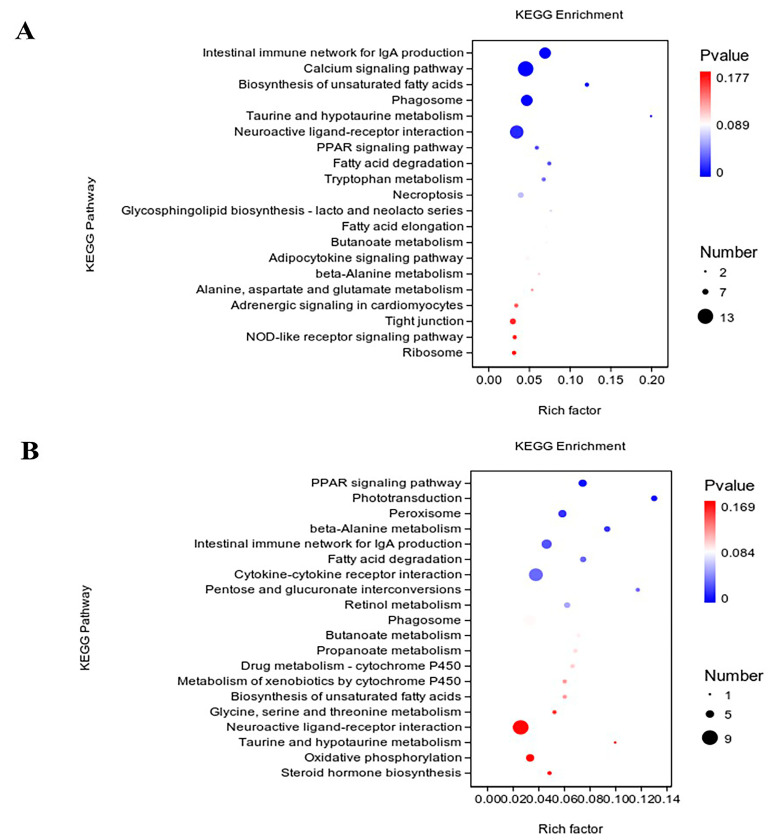
Top 20 KEGG pathways enriched with differentially expressed genes in the ileum and cecum between Shaoxing ducks and Pekin ducks. (**A**) Top 20 enriched KEGG pathways of DEGs identified in the ileum. (**B**) Top 20 enriched KEGG pathways of DEGs identified in the ceca. In the dot plot, the size of each dot reflects the number of genes involved in the corresponding pathway, and the color gradient represents the q-value of the enrichment.

The KEGG enrichment analysis of ileal DEGs is shown in Figure 3A. A total of 76 KEGG pathway terms were identified, of which 9 were statistically significant (*p* < 0.05), including the calcium signaling pathway, biosynthesis of unsaturated fatty acids, and fatty acid degradation. All KEGG enrichment terms identified from the ileal transcriptomes of Pekin and Shaoxing ducks are listed in Table S5.

The KEGG enrichment analysis of ceca DEGs is shown in Figure 3B, also highlighting the top 20 KEGG pathways. A total of 89 KEGG pathway terms were enriched, with 8 significant terms (*p* < 0.05), i.e., PPAR signaling pathway, phototransduction, peroxisome, beta-alanine metabolism, intestinal immune network for IgA production, fatty acid degradation, cytokine–cytokine receptor interaction, and pentose and glucuronate interconversions. All KEGG enrichment terms identified from the cecal transcriptomes of Pekin and Shaoxing ducks are listed in Table S6.

### 3.5. Verification of DEGs by qRT-PCR

**Figure 4:**
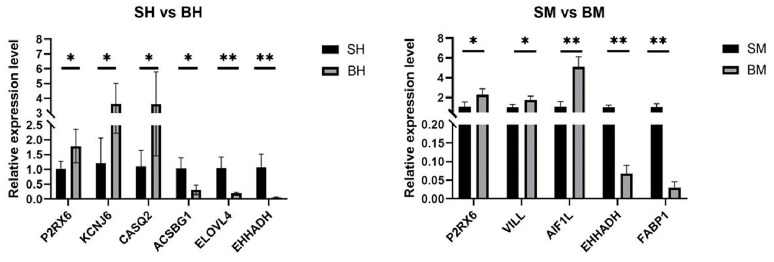
qPCR validation of DEGs in the ileum and cecum of Pekin and Shaoxing ducks. “*” indicates a significant difference (*p* < 0.05); “**” denotes a highly significant difference (*p* < 0.01). SH: ileum of Shaoxing duck; BH: ileum of Pekin duck; SM: cecum of Shaoxing duck; BM: cecum of Pekin duck.

A total of nine putative target genes were chosen for qRT-PCR. These included three genes that were up-regulated in the ileum (*P2RX6*, *KCNJ6*, and *CASQ2*), three genes that were down-regulated in the ileum (*EHHADH*, *ELOVL4*, and *ACSBG1*), three genes that were up-regulated in the ceca (*P2RX6*, *AIF1L*, and *VILL*), and two gene that were down-regulated in the ceca (*EHHADH* and *FABP1*). The veracity of the RNA-seq data was confirmed by the significant variations in these genes’ expression levels and the expression patterns that were found to support the RNA-seq results (Table 3, Figure 4).

## 4. Discussion

Gut development directly influences the digestion, absorption, and metabolism of nutrients, which are crucial for overall health and growth performance [15]. For broiler ducks and laying ducks, growth characteristics are a critical performance indicator, exhibiting significant variation between the two types. In this study, we selected two varieties of high-yielding Shaoxing ducks and high-meat Pekin ducks for transcriptome differences between ileum and cecum. Based on our previous research, the body weight of 42-day-old Pekin ducks notably exceeded that of their Shaoxing counterparts. More importantly, the ileal villus height, as well as the cecal gland depth and mucosal thickness, were significantly greater in Pekin ducks compared to Shaoxing ducks, suggesting that Pekin ducks possess a larger mucosal surface area, thereby enhancing their digestive and nutrient absorption capacities [13]. Numerous studies have demonstrated that altering the intestinal microbiota of poultry can regulate their growth and metabolism [16,17,18,19,20]. However, limited research has been conducted on the impact of the poultry’ s own intestinal tissue on growth and metabolic processes. Our study found that there were slightly more differential genes in the ileum than in the cecum, and there were more differential genes downregulated in the ileum than in the upregulated differential genes, while the ceca were the opposite. This also shows that there are obvious differences between the ileum and cecum of the two Pekin ducks and Shaoxing ducks. We found that GO was mainly enriched in molecular function in the ileum, while biological processes were mainly enriched in the ceca. Key pathways identified in the KEGG enrichment analysis of the ileum and cecum include the calcium signaling pathway, the biosynthesis of unsaturated fatty acids, and fatty acid degradation. This further proves the difference between the intestinal tract of Pekin ducks and Shaoxing ducks and the significant influence on growth and metabolism.

In this study, we also identified several genes implicated in the growth and metabolic processes of Pekin ducks and Shaoxing ducks. Interestingly, at 42 days of age, several genes associated with muscle growth and development are highly expressed in the ileum and cecum of Pekin ducks, whereas genes involved in fatty acid synthesis and metabolism exhibit higher expression in Shaoxing ducks. Our study found that the *P2RX6* gene is significantly enriched in the calcium signaling pathway in KEGG. Moreover, compared to Shaoxing ducks, the *P2RX6* gene is significantly overexpressed in the ileum and cecum of Pekin ducks. The Purinergic receptor P2X, ligand-gated ion channel 6 (*P2RX6*) gene, which is primarily expressed in skeletal muscle, acts as an ATP-gated ion channel receptor and is classified within the purinergic receptor (P2RX) family [21]. The RNA-sequencing analysis indicated that differentiated adipocytes elevated levels of *P2RX6* gene transcripts [22]. Calcium signaling is essential for muscle growth and metabolic regulation, and the higher expression of *P2RX6* in Pekin ducks suggests its potential role in promoting faster growth and greater fat deposition in meat ducks compared to egg ducks. Pekin ducks, as meat ducks, exhibit significantly faster growth and greater fat deposition than Shaoxing ducks. This suggests that the intestinal functions differ between meat ducks and egg ducks and underscores the important role of the intestine in duck growth and development. In this study, Shaoxing ducks have substantially higher levels of *EHHADH* expression in their ileum and cecum than Pekin ducks. The study found that the EHHADH gene is significantly enriched in fatty acid metabolism and peroxisomal lipid metabolism pathways. Enoyl-CoA hydratase and 3-hydroxyacyl CoA dehydrogenase (*EHHADH*) is a protein-coding gene [23]. Some studies have shown that *EHHADH* is highly expressed in the liver [24] and that it is a key gene involved in the fatty acid metabolism pathway in hepatocellular carcinoma [25]. One study showed that 190 metabolites in the kidneys of *EHHADH* knockout mice were significantly altered compared to wild-type mice, with significantly increased levels of 100 of them, including fatty acids and their conjugates [26]. The results imply that *EHHADH* is crucial for lipid metabolism and energy utilization, which could explain its higher expression in Shaoxing ducks, as egg-laying breeds may prioritize lipid metabolism over muscle growth. Therefore, we speculate that the fatty acid metabolism in the ileum and cecum of Shaoxing ducks is significantly higher than in Pekin ducks, further underscoring the important role of the intestine in growth metabolism. In this study, it was found that the expression of calsequestrin 2 (*CASQ2*) and potassium inwardly rectifying channel subfamily J member 6 (*KCNJ6*) were markedly elevated in the ileum of Pekin ducks compared to those in Shaoxing ducks. CASQ2 is a protein-coding gene. Among its associated pathways are cardiac conduction and ion channel transport [27]. *CASQ2* has been implicated in the regulation of muscle growth and development [28]. Calcium signaling plays a fundamental role in muscle contraction, cellular metabolism, and growth regulation. The differential expression of *CASQ2* between the two duck breeds may reflect variations in calcium homeostasis that influence muscle function and overall growth performance. *KCNJ6* is a protein-coding gene. Its related routes include transmission across chemical synapses and inwardly rectifying K^+^ channels. The *KCNJ6* gene has been reported to be linked to egg production in geese [29]. We speculate that the influence of age may result in lower expression of egg production-related genes in Shaoxing ducks compared to Pekin ducks, further indicating that Pekin ducks do indeed grow and develop faster than Shaoxing ducks. According to this study, Shaoxing ducks’ ilea expressed much more ELOVL fatty acid elongase 4 (*ELOVL4*) and acyl-CoA synthetase bubblegum family member 1 (*ACSBG1*) than those of Pekin ducks. *ELOVL4* is a protein-coding gene involved in pathways such as fatty acyl-CoA biosynthesis and metabolism. This gene encodes a membrane-associated protein that is a part of the ELO family, a group of proteins involved in fatty acid biosynthesis. *ACSBG1* is a protein-coding gene. Its associated pathways include fatty acid metabolism and arachidonate biosynthesis III (metazoa). *ELOVL4* was found to be associated with polyunsaturated fatty acids (PUFAs) synthesis in chickens [30]. *ACSBG1* was found to be associated with fatty acid metabolism and degradation in chickens [31]. At the same time, we also found that the allograft inflammatory factor 1-like (*AIF1L*) and villin (*VILL*) genes, which are associated with muscle development, were highly expressed in the ceca of Pekin ducks, whereas the fatty acid-binding protein 1 (*FABP1*) gene was highly expressed in Shaoxing ducks. *AIF1L* and *VILL* genes promote actin binding [32], and VILL is a component of the cytoskeleton [33]. *FABP1* is a fatty acid-binding protein, which has a role in the short uptake, transport, and metabolism of fatty acids and participates in intracellular lipid transport [34]. We speculate that due to the faster growth and development of Pekin ducks compared to Shaoxing ducks, genes associated with muscle growth is highly expressed in the ileum and cecum of Pekin ducks, whereas those related to fatty acid metabolism are expressed at lower levels than in Shaoxing ducks.

In summary, we characterized and evaluated the ileal and cecal transcriptomes of Pekin ducks and Shaoxing ducks. Notable differences exist between the ileum and cecum of Pekin ducks and Shaoxing ducks. Comprehensive analysis revealed that the genes *P2RX6*, *CASQ2*, *KCNJ6*, *AIF1L*, and *VILL* contribute to the regulation of growth and development of Pekin and Shaoxing ducks, whereas *EHHADH*, *ELOVL4*, *ACSBG1*, and *FABP1* participate in lipid production and metabolic processes within these breeds. Our results suggest that the apparent variations in gene expression can be used as a foundation for more study on this subject. Future research could focus on validating the roles of these identified genes in growth and metabolism through functional experiments, such as gene knockout or overexpression studies, or through genetic knockdown approaches. Additionally, more studies could explore the molecular mechanisms underlying the differences in intestinal function between Pekin and Shaoxing ducks, including the role of calcium signaling and other metabolic pathways. It is also crucial to consider the impact of environmental and dietary factors on gene expression, as these elements can significantly influence gene activity and overall metabolic regulation. This would significantly advance our understanding of the genetic and metabolic regulation in these two duck breeds, ultimately contributing to better breeding and management strategies.

## 5. Conclusions

In conclusion, the ileal tissue included six genes linked to growth and metabolism, namely, *P2RX6*, *KCNJ6*, *CASQ2*, *EHHADH*, *ACSBG1*, and *ELOVL4*. The cecal tissue also included five genes linked to growth and metabolism, namely, *P2RX6*, *KCNJ6*, *AIF1L*, *VILL*, *FABP1*, and *EHHADH*. Among these genes, those associated with calcium metabolism and skeletal muscle synthesis exhibited higher expression levels in Shaoxing ducks, whereas genes related to fatty acid synthesis and transport were more highly expressed in Pekin ducks. This study provides a theoretical basis for exploring the effects of ileum and cecum on the growth and metabolism of ducks, fills the knowledge gap on the differences in the ileal and cecal tissues between meat ducks and laying ducks, and provides a basis for further research on the genes affecting the growth and metabolism of ducks. However, potential confounding factors such as diet, environmental conditions, and specific breed traits should be acknowledged as they may also influence gene expression and metabolism. Future research could focus on validating the functional roles of these genes through in vitro approaches, such as CRISPR-based knockdown or knockout experiments, as well as in vivo studies to directly examine their impact on growth and metabolism. Such studies would offer deeper insights into the molecular mechanisms underlying duck growth and metabolism and could inform breeding strategies for both meat and egg production.

## Figures and Tables

**Table 1:** Primers for RT-qPCR.

Gene Name	Product Size (bp)	TM (°C)	Amplification Efficiency	Primer Sequences (5′ to 3′)
GAPDH	99	60	100%	F: TCTGGCAAAGTGGAAGTGGT R: CCGGAAGTGGCCATGAGTAG
P2RX6	78	60	100%	F: CAAACCAACGACTCCACCTA R: ATGCTGATGCTTCCTCCC
KCNJ6	82	60	100%	F: TGACATGCCAAGCTCGAAGT R: TCCTCCAGAGTGAGGACAGG
CASQ2	98	60	100%	F: CCAGGAGCATCGGGTTTG R: TTTGGCGAGTTTGGCATC
ACSBG1	60	60	100%	F: TCCAGGAAAGCAGCCAAGAG R: TGCTACGCTGTGGAATCGTT
EHHADH	207	60	100%	F: GCTTGGGCCCCTTAGTTTCT R: CGATCAGTCTGGGTAGTCGC
ELOVL4	67	60	100%	F: CTCGAGTTCTACCGCTGGAC R: ACTGCATCAAGGGCCAATCA
AIF1L	61	60	100%	F: TCTGTGACCCGAAGTTCAGTG R: TCTTTGAACACCGCCAGCTT
VILL	65	60	100%	F: CAAAACTGCCAAGGTGGAGC R: CTAGCTCTGGTGTCGCATGC
FABP1	66	60	100%	F: CTGAAGGGGCTGAAATCCGT R: TCCCATGGTCATTGTGTGGG

**Table 2:** Summary of reads and matches.

Samples	Read Numbers	Clean Reads	Clean Bases	Clean Ratio (%)	Q30 (%)
BJ_1H	49915050	46955144	7090226744	94.07	92.74
BJ_2H	44497058	42069004	6352419604	94.54	92.75
BJ_3H	44070764	41686758	6294700458	94.59	92.18
BJ_4H	55879402	52890360	7986444360	94.65	93.21
BJ_5H	43124648	40771040	6156427040	94.54	92.44
SX_1H	51437022	48631200	7343311200	94.55	92.03
SX_2H	46469116	43974506	6640150406	94.63	92.60
SX_3H	42176790	39892012	6023693812	94.58	93.03
SX_4H	50527934	47843848	7224421048	94.69	92.96
SX_5H	43820670	41437276	6257028676	94.56	93.04
BJ_1M	45677740	43254128	6531373328	94.69	93.00
BJ_2M	42250402	39929594	6029368694	94.51	93.09
BJ_3M	50843934	48039702	7253995002	94.48	92.46
BJ_4M	46317106	43723224	6602206824	94.40	92.18
BJ_5M	45838652	43283748	6535845948	94.43	92.34
SX_1M	43444586	41150334	6213700434	94.72	91.01
SX_2M	50425364	47622128	7190941328	94.44	91.96
SX_3M	47810534	45235762	6830600062	94.61	92.46
SX_4M	49549788	46838862	7072668162	94.53	91.62
SX_5M	48650494	45987318	6944085018	94.53	92.46

**Table 3:** List of partially representative DEGs.

Organization	Different Expression Type	Genes	Description	log_2_FoldChange	*p*-Value
Ileum	Up-regulated	P2RX6	Purinergic receptor P2X, ligand-gated ion channel 6	1.620	0.017
		KCNJ6	Potassium Inwardly Rectifying Channel Subfamily J Member 6	2.241	0.019
		CASQ2	Calsequestrin 2	2.673	0.010
	Down-regulated	EHHADH	Enoyl-CoA Hydratase And 3-Hydroxyacyl CoA Dehydrogenase	1.152	0.003
		ACSBG1	Acyl-CoA Synthetase Bubblegum Family Member 1	1.727	0.025
		ELOVL4	ELOVL Fatty Acid Elongase 4	1.733	0.003
Cecum	Up-regulated	P2RX6	Purinergic receptor P2X, ligand-gated ion channel 6	1.979	0.029
		AIF1L	Allograft Inflammatory Factor 1 Like	1.314	0.001
		VILL	Villin Like	1.564	0.014
	Down-regulated	EHHADH	Enoyl-CoA Hydratase And 3-Hydroxyacyl CoA Dehydrogenase	1.789	0.000
		FABP1	Fatty Acid Binding Protein 1	1.400	0.006

## Data Availability

The original data have been uploaded to NGDC under project ID PRJCA38056 (https://ngdc.cncb.ac.cn/, accessed on 1 April 2025).

## Supplementary Materials

The following supporting information can be downloaded at https://www.mdpi.com/article/10.3390/genes16050488/s1: Table S1: DEGs in the ileum between Shaoxing ducks and Pekin ducks; Table S2: DEGs in the ceca between Shaoxing ducks and Pekin ducks; Table S3: GO enrichment analysis of ileum DEGs between Shaoxing ducks and Pekin ducks; Table S4: GO enrichment analysis of ceca DEGs between Shaoxing ducks and Pekin ducks; Table S5: KEGG enrichment analysis of ileum DEGs between Shaoxing ducks and Pekin ducks; Table S6: KEGG enrichment analysis of ceca DEGs between Shaoxing ducks and Pekin ducks; Figure S1: RNA-Seq correlation check.
