# Supplementary material for: Comparative Transcriptome Profiling of Ileal and Cecal Tissues Between Pekin Ducks and Shaoxing Ducks
Source: Genes (Basel). 2025 Apr 25;16(5):488. doi: 10.3390/genes16050488 (PMC12110802; doi:10.3390/genes16050488)
Supplement: Supplementary file 1 [file genes-16-00488-s001.zip › Figure S1 RNA-Seq correlation check .pdf]

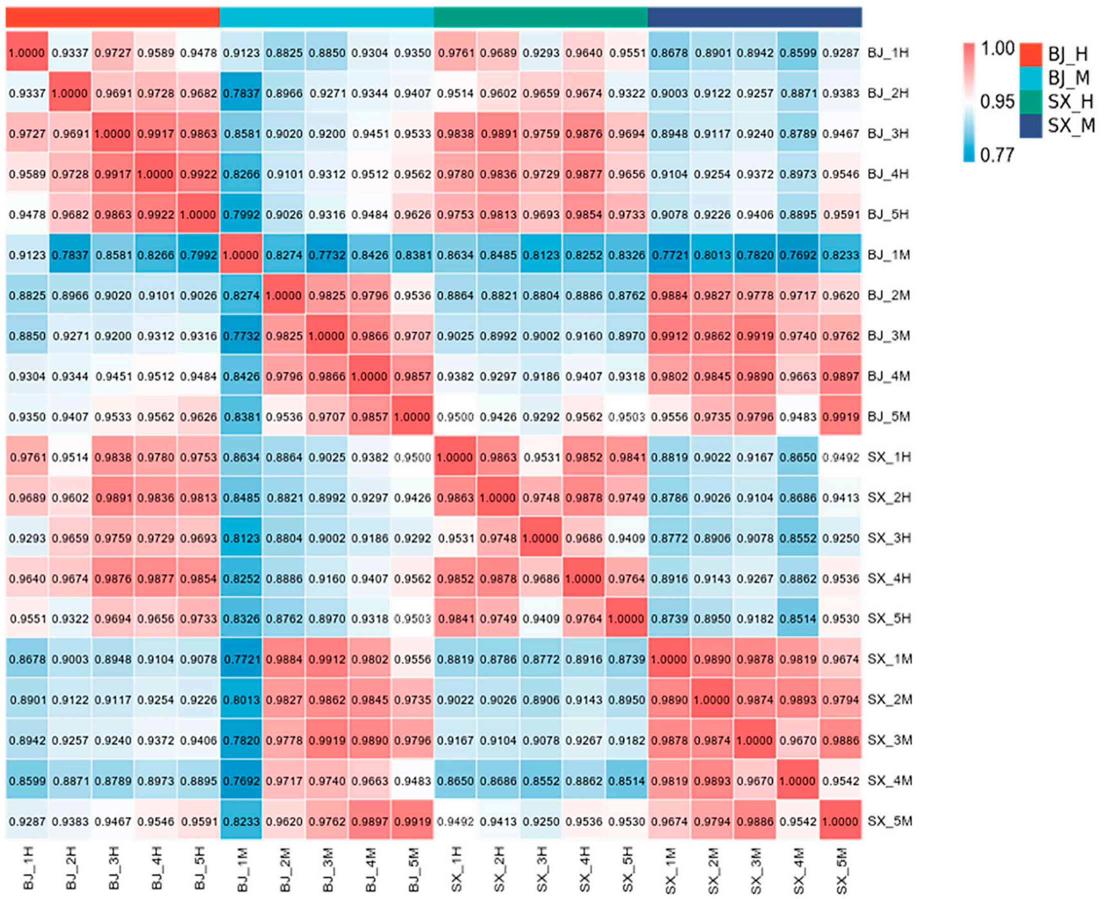

Figure S1. RNA-Seq correlation check. BJ_1H ~ BJ_5H is the ileum sample of Pekin ducks, and BH_1M ~ BH_5M is the cecal sample of Pekin ducks. SX_1H ~ SX_5H is the ileum sample of Shaoxing ducks, and SX_1M ~ SX_5M is the cecal sample of Shaoxing ducks.
